# Selectively Blocking Small Conductance Ca^2+^-Activated K^+^ Channels Improves Cognition in Aged Mice

**DOI:** 10.3390/biology14020149

**Published:** 2025-02-01

**Authors:** Jessie Ong, H. Craig Heller, Elsa Pittaras

**Affiliations:** Department of Biology, Stanford University, Stanford, CA 94305, USA; jessieo@stanford.edu (J.O.); hcheller@stanford.edu (H.C.H.)

**Keywords:** apamin, K^+^ channel, memory, motor skills, motivation, risk taking, anxiety

## Abstract

Aging is associated with decreased neuronal sensitivity and activity that creates deficits in cognitive processes, including learning, memory, motivation, general activity, and other behaviors. These effects are due in part to decreased intracellular Ca^2+^ homeostasis, increasing hyperpolarization of the resting potential in aged neurons and therefore decreasing their excitability. To reduce hyperpolarization in aged mice, we used apamin, a selective small conductance Ca^2+^-activated K^+^ (sK_Ca_) channel blocker. By blocking the sK_Ca_ channels, apamin decreases the egress of the K^+^ out of the cell, reducing its hyperpolarization and causing it to be closer to threshold potential. As a result, neurons should be more sensitive to excitatory stimuli and more active. We evaluated the performance of aged mice in a selection of cognitive and behavioral tests prior to and after systemic applications of apamin or the vehicle saline. Apamin improved performance in short-term memory, increased attention to tasks, and decreased anhedonia. Apamin had no significant effect on long-term spatial and recognition memory, risk-taking behavior, sociability, and anxiety. Our results are compatible with the known effects of sK_Ca_ channel blockade on neuronal sensitivity and activity; however, these short-term effects were not reflected in longer-term alterations of neural plasticity responsible for long-term spatial and recognition memory or other more complex cognitive processes we evaluated.

## Simple Summary

Neural activity decreases with age and therefore may be causative in age-related decrements in cognition. A major negative controller of neural activity is calcium-activated small conductance potassium channels. These ion channels are blocked by the apamin molecule, which is a component of bee venom. We show that the treatment of aged mice with apamin increases some cognitive behaviors, but not all.

## 1. Introduction

The number of individuals aged 60 and older in the world is expected to grow by 38%, from 1 billion to 1.4 billion, in the next 10 years [1]. Aging is associated with cognitive decline. A 2022 report from Columbia University found that the incidence of dementia in the population over 65 is about 10%, and the incidence of mild cognitive impairment is an additional 22%. Thus, the disease burden of cognitive impairment is huge and will continue to grow. The need to understand underlying causes and develop mitigating therapies is critical.

Functional decline is seen in many nervous system domains, including learning, memory, analytical and abstract thinking, motivation, and activity. Therefore, there are likely to be causes that go beyond a particular neuronal mechanism, such as a specific circuit or transmitter/receptor relationship. Overall cognitive decline is likely to involve neuronal processes that are common to all or most neurons. One such general function is the maintenance of neuronal membrane resting potentials, which determine cell activities and sensitivity to stimuli. More negative resting potentials put the cell farther from the threshold for firing action potentials in response to synaptic input or in response to cell autonomous factors. The cell’s resting membrane potential is determined by the movement of ions into and out of the cell by transporters or through channels driven by concentration gradients and the permeability of the channels. A critical ion determining the resting potential is potassium (K^+^). K^+^ moving out of the cell through K^+^ channels down its concentration gradient leaves the cell with a more negative charge and farther from its firing threshold. There are many types of K^+^ channels [2]. Leak channels are constitutively open and are the most important contributors to the resting potential. Voltage gated channels respond to transmembrane potential and contribute to repolarization following an action potential. Calcium-activated small conductance K^+^ channels (sK_Ca_ channels) respond to changes in Ca^2+^ and are the focus of this study. There are three subtypes of sK_Ca_ channels [3]. One reason for suspecting sK_Ca_ channels in aging-related cognitive decline is that aging has been shown to impair intracellular Ca^2+^ homeostasis, elevating intracellular Ca^2+^ and increasing K^+^ movement from the cell and hyperpolarizing the membrane [3,4]. Moving the membrane resting potential farther from threshold makes it less responsive and impairs functions such as neuroplasticity.

Studies have shown that sK_Ca_ channels play an important role in modulating neuroplasticity and therefore learning and memory [5,6,7,8]. Blank et al. [9] showed that overexpression of sK_Ca_2 channels in the hippocampus of aged mice reduces their long-term potentiation and impairs their performance in a learning task. They suggested that selectively reducing the function of these channels might be a possible mechanistic approach for pharmacological treatments to ameliorate aging-related memory deficits. One approach to reduce the K^+^ conductance of the sK_Ca_ channels is application of the toxin apamin, which occurs naturally in the venom of honeybees [10]. Apamin is highly selective and only blocks sK_Ca_ channels. The treatment of mice and rats with low doses of apamin improves their performance in several rodent learning tasks [5,6,7]. Since most experiments on the effects of apamin on rodent learning and memory have been done on young or midlife subjects, we thought it would be informative to investigate the effects of sK_Ca_ blockade on aged mice as an indication of whether such pharmacotherapy might be feasible for treatments of cognitive decline in the elderly population. 

We administered apamin intraperitoneally (IP) in aged mice and evaluated cognition and behavior. We hypothesized that apamin would result in increased intracellular [K^+^], a less negative resting membrane potential, and increased neuronal excitability. We hypothesized that apamin could improve deficits in cognition, motivation, and locomotion of the aged mice. To test our hypothesis about the effects of apamin, we administered the following tests: open field, novel object recognition and location (NOR and NOL), T-Maze, nesting, social partitioning, elevated plus maze, and sucrose preference at baseline, with a vehicle, and with apamin.

## 2. Materials and Methods

### 2.1. Animals

All experiments were performed in accordance with the guidelines described in the National Institutes of Health Guide for the Care and Use of Laboratory Animals, and all procedures were approved by the Stanford University Institutional Animal Care and Use Committee and are in accordance the ARRIVE Guideline (2025, https://arriveguidelines.org/arrive-guidelines). Efforts were made to minimize the number of animals used and the pain and distress they experienced. All experiments were randomized: half of the mice received the saline injection first and then the apamin, and for the other half the order of the injections was reversed. This approach was used to prevent potential bias due to the sequencing of the experimental ordering. 

Wildtype male mice between the ages of 16 and 21 months, Ts(17<16>)65Dn mice, obtained from Jackson Laboratories, Sacramento, CA. were used in this study. Those mice were also tested at young ages (5 months old) in the T-maze and the NOR. Mice were housed in groups of 2–4 per cage in a 22 °C and 40–60% humidity-controlled room. The light cycle was 12 h light/12 h dark, with lights on at 8:00 am. Food and water were available ad libitum, except during the sucrose preference test, for which mice were water deprived for a night. Most of the mice used in these experiments completed the NOR and the T-maze at a younger age (5 months) but not the other behavioral tests.

**Figure 1:**
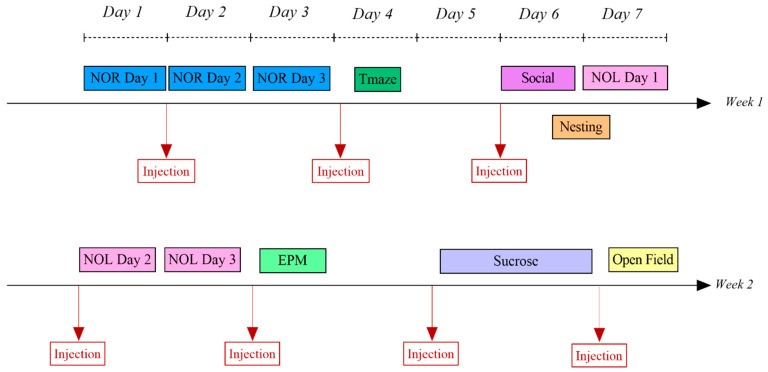
Experimental timeline overview. Injections were performed 30 min before each test. The same timeline was followed for the baseline before these two weeks of behavioral experiments with injections of saline or apamin.

Prior to any systemic injections, baseline learning, memory, and behavior were evaluated. All cognitive and behavioral tests were conducted at the end of the light phase and in different environments with different objects to prevent mice from remembering previous iterations of the same test. Two randomized 2-week sessions were performed: one with saline injections and one with apamin injections. Mice were used as their own matched controls between baseline, saline, and apamin trials. Figure 1 describes the order of the behavioral testing during one two-week session.

### 2.2. Apamin and Saline Administration

**Figure 2:**
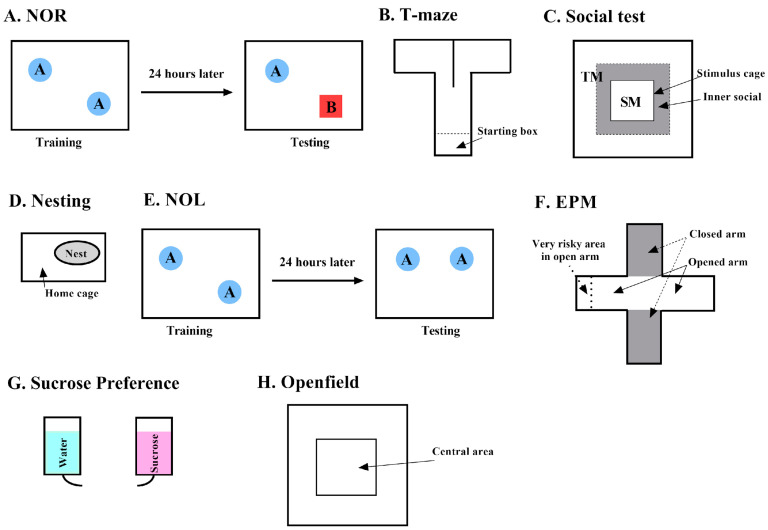
Schematic representation of the behavioral test: The Novel Object Recognition (NOR, (**A**)), T-maze (**B**), Social test (**C**), Nesting test (**D**), Novel Object Location (NOL, (**E**)) Elevated Plus Maze (EPM, (**F**)), Sucrose Preference test (**G**), and Openfield (**H**). TM = tested mouse, SM = stimulus mouse.

Apamin and saline injections were administered, intraperitoneally, at 0.1 mg/kg [11]. As shown in Figure 2, all injections were performed 30 min before the behavioral test (for the Open Field, Partition, and T-Maze) or before being housed alone (for the nesting and sucrose tests). For the NOR and NOL tests, injections occurred 30 min before the training session (Day 2, Figure 1).

### 2.3. Behavioral Tests

#### 2.3.1. Novel Object Recognition (NOR)

The objective of the NOR is to assess long-term recognition memory. On the first day, mice were habituated to an empty arena made of white plastic (50 × 50 × 50 cm, Figure 2A). On the second training day, two identical objects were placed in the empty arena at the same distance from the wall and corners of the arena. Mice were allowed to explore both objects for 10 min. If mice spent more than 75% or less than 25% of the time with one object over the other, this mouse was eliminated from the data set to avoid any preference or aversion for an object and/or any lack of sufficient exploration during the training session. The following day, testing day, one of the objects was replaced by a novel object. Mice were allowed to explore these two objects for 10 min. Mice who spent more time with the novel object demonstrated long-term recognition memory.

The mouse’s behavior was recorded by an infrared-sensitive camera placed 2.5 m above the arena. The Viewpoint VideoTrack system (Montreal, QC, Canada) recorded the time spent within a 2 cm distance within each object during both the testing and training days. We used the following formula to determine the discrimination index of the novel object compartment to the unchanged object.

$$DI\;score=\frac{Time\;novel-unchanged\;object}{Time\;novel+unchanged\;object}$$

This test was conducted when the mice were young (5 months old) and old (16 to 21 months old).

#### 2.3.2. T-Maze

The T-maze evaluates short-term spatial memory by allowing mice to choose between two arms of a T-shaped maze. The frequency of their alternations in exploring the two arms of the T is taken as a measure of their short-term memory ability. Spontaneous alternation is based on the natural tendency of rodents to consecutively alternate between left and right arm choices during exploration in a T-maze apparatus. For the first 30 s, mice are confined in a start box (15 × 7 × 20 cm) in the stem arm of the transparent, plastic T-maze (48 × 7 × 20 cm, Figure 2B). The start box is separated from the rest of the maze by a transparent door that is lifted at the end of this 30 s. After the door is open, mice are allowed to freely explore the T-maze for 7 min. At the center of the T-Maze intersection, a dividing wall (8.3 cm) separates the stem into the two arms to force mice to decide between left and right arms. Entrances into the left or right arms are recorded manually for a duration of 7 min. An entrance is scored when at least 2/3 of the body of the mouse has entered an arm. Alternations are scored when a mouse makes an entrance into a left or right arm and then enters the opposite arm. A reentry into the same arm is not scored as an alternation. Percent alternations were calculated with the following formula:

$$\%\;Alternations=\frac{Number\;of\;alternations}{Number\;of\;entrances}\times 100$$

A mouse was excluded from the data set if the percentage of alternations was <25% or the total number of entrances was less than 6. The apparatus was cleaned with 10% ethanol, dried, and ventilated for a few minutes between mice.

This test was conducted when the mice were young (5 months old) and old (16 to 21 months old).

#### 2.3.3. Sociability Test

The Social Test is designed to detect autistic-like or anti-social behaviors by evaluating the interactions of the test mouse (TM) with a stimulus mouse (SM). In a testing arena (50 × 50 × 50 cm), a stimulus cage (10 × 10 × 15 cm) is placed in the center (Figure 2C). The stimulus cage is transparent and perforated to allow visual, olfactory, and auditory cues between mice. An SM is placed inside the stimulus cage. The TM is placed in the testing arena and allowed to explore for 10 min. Between test rounds, the stimulus cage and test arena are cleaned with 10% ethanol. Tests are performed in a quiet, dim room to minimize external stressors. Using Viewpoint software, version 3.52, we defined a Social Zone (SZ) within the testing arena as the area within 8 cm of the walls of the stimulus cage. We tracked the time spent in the SZ (in seconds) and the number of entrances into the SZ. A Sociability Index (SI) was calculated with the following formula:

$$\%\;SI=\frac{Time\;spent\;in\;SZ-Time\;spent\;in\;non-SZ}{Time\;spent\;in\;the\;all\;area}\times 100$$

Mice with a higher SI spend more time in the social zone and are considered to exhibit normal sociability. Mice with a lower SI travel shorter distances in the SZ and have fewer nose touches and spend less time on top of the stimulus cage, indicative of antisocial behavior and potentially reflecting autistic-like traits.

#### 2.3.4. Nesting Test (Motivated Activity)

The nesting test was used to evaluate motivated activity using a 5-point rating scale [12]. One hour before the dark phase, mice were transferred from group to individual testing cages. One nestlet of 2.5 g was placed in each cage. Mice spent 12 h in individual housing (Figure 2D). Nesting scores were blindly evaluated to avoid potential biases. A score of 1–5 was given based on the following parameters:

Score 1: Nestlet is more than 90% intact.
Score 2: Nestlet is 50–90% intact.
Score 3: Nestlet is 50–90% shredded but cotton is not gathered in a nest.
Score 4: Nestlet is more than 90% shredded and cotton is gathered in a nest. The nest is flat.
Score 5: Nestlet is more than 90% shredded and cotton is gathered in a nest. The nest forms a crater.

#### 2.3.5. Novel Object Location (NOL)

The objective of NOL is to assess long-term spatial memory through a three-part test: habituation, training, and testing. It is performed in the same way as the NOR; however, on the testing day, one of the identical objects is moved to a different location instead of being replaced by a new object (Figure 2E). A discrimination index was calculated with the formula below:

$$DI\;score=\frac{Time\;object\;at\;the\;new\;location-unchanged\;object}{Time\;object\;at\;the\;new\;location+unchanged\;object}$$

As with the NOR, mice that spent more than 75% or less than 25% of the time with one object over the other during training day were eliminated from the data set to avoid any preference or aversion to a particular object and/or any lack of sufficient exploration during the training session.

#### 2.3.6. Elevated Plus Maze (EPM)

EPM assesses anxiety and risk-taking behaviors in mice. The EPM is an elevated maze (75 cm above ground) with two wall-enclosed arms (30 × 5 × 25 cm) and two open arms (30 × 5 cm). All four arms are connected by a central platform (5 × 5 cm, Figure 2F). A mouse is placed in the central platform facing both an enclosed arm and an open arm and is allowed to explore for 7 min.

The following parameters were used to quantify anxiety and risky behavior: number of entrances and time spent in open arms (when both front paws are placed in the open arm), number of head dips in open arms (when the head of the mouse is bent over the border of the open arms), and time and entrances into the very risky zone (when both front paws are within the end 5 cm of the open arms). The apparatus was cleaned with 10% ethanol, dried, and ventilated for a few minutes between mice.

#### 2.3.7. Sucrose Preference Test (SPT)

SPT measures anhedonia, a core symptom of depression, by evaluating the preference for a sweet sucrose solution over water over a 12 h test period (6 am–6 pm, Figure 2G). To avoid any novelty aversion for a new solution containing 1% sucrose, for a period of three days, group-housed mice were provided with two bottles containing a 1% sucrose solution instead of water. Mice were water-deprived for one night before the start of the experiment. After the water deprivation period, mice were placed in individual cages and provided with two bottles: one containing 1% sucrose solution and the other containing water. For the first 6 h, a random number generator was used to assign the initial position of the sucrose bottle (left or right side of the cage). At 6 h into the test (12 pm), the positions of the sucrose solution and water bottles were swapped to counteract any side preference/aversion bias. After the 12 h test period, the final weight of each bottle was recorded. The volume of sucrose solution and water consumed was calculated by subtracting the final weight from the initial weight of each bottle. Sucrose Preference (SP) was calculated by dividing the volume of sucrose consumed by the total volume of fluid consumed (sucrose + water) and multiplied by 100.

$$\%\;Sucrose\;Preference=\frac{Volume\;sucrose\;consumed}{Volume\;water+sucrose\;consumed}\times 100$$

High SP indicates normal hedonic behavior, while low SP is suggestive of anhedonia and depressive-like behavior.

#### 2.3.8. Open Field Test

Dring the open field test, mice are put in the middle of a large arena (50 × 50 × 50 cm), resting on an infrared emitting base, for 10 min. We measure the time the animal is spending in the central area of this open field. Because anxious, fearful mice usually avoid open spaces and tend to stay close to walls, the more the mouse spends time in the center of the open field, the less the mouse is showing anxiety-like behavior. The mouse’s behavior was recorded by an infrared-sensitive camera placed 2.5 m above the arena. The inner zone (IZ, 10 × 10 cm, Figure 2H) at the center of the Openfield was created using Viewpoint software. Total time and number of entrances into the IZ were recorded by the software. IZ index, the time spent in IZ divided by total time spent in test arena, was calculated with the following formula:

$$\%\;Inner\;Zone=\frac{Time\;in\;IZ}{Total\;time\;in\;testing\;area}\times 100$$

More anxious mice have decreased IZ indices, as they do not explore the central area and spend more time along the walls and in the corners. Data were analyzed using Video track software from ViewPoint Life Sciences, Inc. (Montreal, QC, Canada). The apparatus was cleaned with 10% ethanol, dried, and ventilated for a few minutes between mice.

#### 2.3.9. Statistical Analyses

Statistical analyses were performed using Statview software, Version 4.57. If the data showed normal distribution (Shapiro–Wilk test) and passed equal variance tests (*F* test), statistical analyses were performed using *t*-tests or analysis of variance (ANOVA). If not, statistical analyses were performed using Wilcoxon task sign-rank, Kruskal–Wallis or Mann–Whitney non-parametric tests. For the behavioral tests, ANOVAs were used to test the effect of treatment, time (training vs. testing), and the combined effect of treatment × time as independent values. For multiple comparisons, a post hoc correction was applied. Results were reported as means ± SEM. *p* values ≤ 0.05 are considered statistically significant.

## 3. Results

### 3.1. Memory Tests

#### 3.1.1. T-Maze

**Figure 3:**
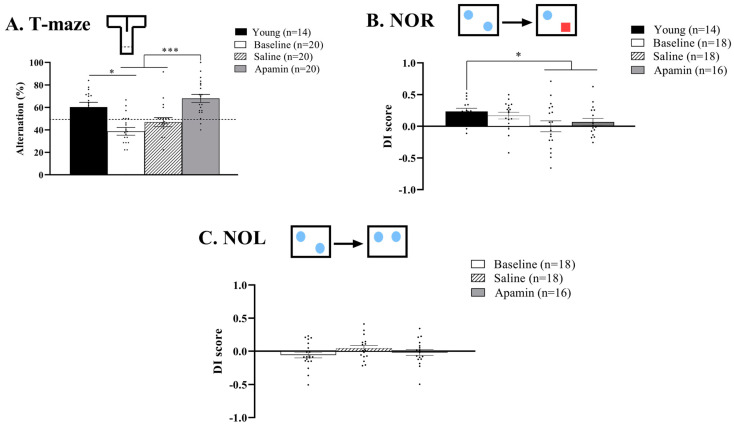
**Effect of apamin treatment on memory in mice.** Apamin improves spatial short-term memory in aged mice (**A**). Long-term memory evaluated by percentage duration with the novel object in NOR (**B**) and the object at the new location in the NOL test. Red square represents novel object, blue square are representative of habituated objects (**C**). T-test, * *p* < 0.05, and *** *p* < 0.001, significant difference between young, baseline, saline and/or apamin. Blue squares represent habituated objects.

The repeated measurement ANOVA showed the effects of treatment on short-term memory (F(2,38) = 25.830, *p* < 0.0001, Figure 3A). Indeed, apamin treatment led to an increase of the percentage of alternation compared to the baseline (from 39% to 68%, t = −6.104, *p* < 0.0001) and to the saline injection (from 47% to 68%, t = −5.570, *p* < 0.0001). The saline injection did not improve short-term memory from the baseline level (t = −2.060, *p* = 0.05). A significant difference was observed between the young mice and the baseline for the older mice (t = 3.528, *p* = 0.003), but this difference was not significant after the saline (t = −1.612, *p* = 0.13) or apamin injection (t = 1.678, *p* = 0.12).

#### 3.1.2. NOR

The repeated measurement ANOVA did not show the effects of treatment on long-term recognition memory (baseline 0.17, saline 0.01, and apamin 0.1; F(2,24) = 2.986, *p* = 0.06, Figure 3B). Compared to their performance when they were young, mice showed a significant impairment of their recognition memory after apamin or saline injection but not compared to the baseline (F(3,27) = 3.795, *p* = 0.02; young vs. baseline: t = −0.010, *p* = 0.99; young vs. saline: t = 4.851, *p* = 0.0004; young vs. apamin: t = 2.327, *p* = 0.04).

#### 3.1.3. NOL

The repeated measurement ANOVA showed no effects of the treatment on long-term spatial memory (F(2,26) = 1.367, *p* = 0.3, Figure 3C).

### 3.2. Depressive-Like Behavior Tests

#### 3.2.1. Sucrose

**Figure 4:**
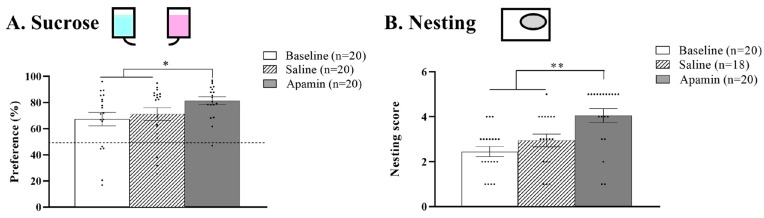
**Effect of apamin on depressive-like behavior.** (**A**) Mean sucrose preference at baseline and after saline or apamin injections. Blue representative of water and pink representative of sucrose solution (**B**) Mean nesting scores at baseline and after saline or apamin injections. T-test, * *p* < 0.05, ** *p* < 0.01 significant difference between baseline, saline, and/or apamin.

The repeated measurement ANOVA showed the effects of treatment on the percentage of sucrose preference (F(2,36) = 3.910, *p* = 0.02, Figure 4A). Indeed, apamin treatment led to an increase of sucrose preference compared to the baseline (from 67% to 81%, t = −2.906, *p* = 0.009) and to the saline injection (from 71% to 81%, t = −2.518, *p* = 0.02). The saline injection did not improve sucrose preference from the baseline level (t = −0.602, *p* = 0.55).

#### 3.2.2. Nesting

The repeated measurement ANOVA showed the effects of treatment (F(2,34) = 15.275, *p* < 0.001, Figure 4B). Indeed, apamin treatment led to an increase in the nesting score of mice from the baseline (from 2.45 to 4.05, Z = −3.408, *p* = 0.007). After saline injection, the nesting score of the mice did not improve from the baseline (from 2.45 to 2.9, t = −1.810, *p* = 0.08). However, the nesting score was improved after apamin compared to saline injection (Z = −2.769, *p* = 0.006).

### 3.3. Social Tests

**Figure 5:**
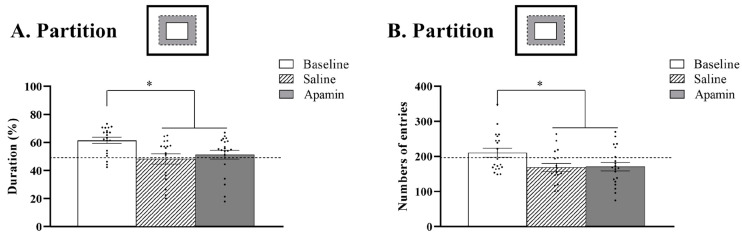
**Effects of apamin on social behavior in aged mice.** Sociability was quantified by (**A**) seconds spent in the social zone and (**B**) number of entries into the social zone. T-test or Wilcoxon test, * *p* < 0.05 significant difference between baseline (*n* = 20), saline (*n* = 17), and/or apamin (*n* = 20).

The repeated measurement ANOVA showed the effects of treatment on the time spent in the social area (F(2,32) = 4.188, *p* = 0.02, Figure 5A) and the number of entries in the social area (F(2,32) = 4.815, *p* = 0.01, Figure 5B). Indeed, apamin treatment led to decreased time spent in the social zone (from 61% to 51%, Z = −2.632, *p* = 0.008) and a decreased number of entries in the social area (210 to 171 entries, t = 2.135, *p* = 0.04) from the baseline. After saline injection, the time spent in the social zone of the mice also decreased (from 61% to 48%, t = 2.401, *p* = 0.03) as well as the number of entries (from 210 to 168 entries, t = 2.302, *p* = 0.03). No difference was observed between the saline and the apamin injection regarding the time spent in the social area (Z = −0.071, *p* = 0.94) as well as the number of entries in the social area (t = −0.547, *p* = 0.6).

### 3.4. Risk Taken, Anxiety Tests

#### 3.4.1. Openfield

**Figure 6:**
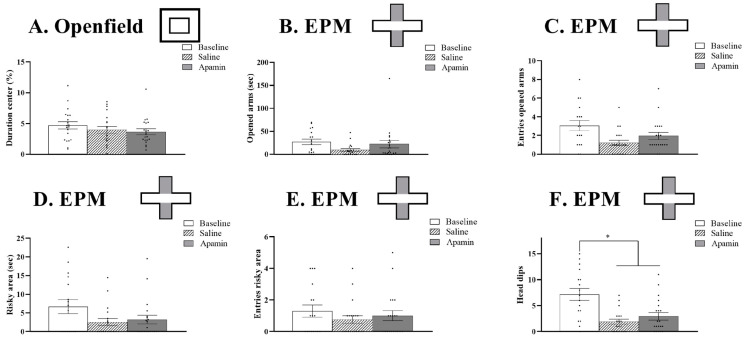
Effects of apamin on anxiety and risk taking. Risky decision making was quantified by the percentage of time spent in the central area of the Openfield (**A**), the duration and number of entries in the open arm of EPM (**B**,**C**) and in the risky zone of EPM (**D**,**E**) as well as the number of head dips in EPM (**F**). T-test or Wilcoxon test, * *p* < 0.05 significant difference between baseline (*n* = 19), saline (*n* = 17), and apamin (*n* = 19).

The repeated measurement ANOVA did not show any effects of treatment on the percentage of time spent in the central area (baseline 4.6%, saline 3.9%, and apamin 3.6%; F(2,38) = 1.670, *p* = 0.20, Figure 6A).

#### 3.4.2. EPM

Apamin injections had no effect on the time and the number of entries in the open arms (time: F(2,30) = 2.476, *p* = 0.1; entries: F(2,30) = 2.305, *p* = 0.12, Figure 6B,C) as well as in the very risky area (time: F(2,30) = 2.525, *p* = 0.09; entries: F(2,30) = 1.446, *p* = 0.25, Figure 6D,E). However, repeated measurement ANOVA showed the effects of treatment on the number of head dips (F(2,30) = 8.150, *p* = 0.0015, Figure 6F). This effect was due to a significant decreased number of head dips between the baseline and the saline injection (t = 3.589, *p* = 0.02) as well as the apamin injection (t = 2.728, *p* = 0.01). No differences were observed between the saline and the apamin injection (t = −1.085, *p* = 0.3).

## 4. Discussion

### 4.1. Mechanism of SK Channels

We explored the roles of small conductance, calcium-activated K^+^ (sK_ca_) channels in age-related cognitive deficits. sK_ca_ channels may be involved in cognitive deficits regardless of their ultimate cause: aging, genetics, disease, or other factors. Learning and memory are a major focus for studies of the neurobiology of cognition, but cognition involves behaviors that are not strictly learning and memory. For example, attention, motivation, recognition of risk, sensory evaluation, social behavior, and emotional reactions are all components of cognition. They all involve changes in neuronal excitability and activity. sK_ca_ channels are significant controllers of membrane resting polarity, which influences excitability. There are three subtypes of sK_ca_ channels, SK1, SK2, and SK3 [2,3]. Apamin is a selective blocker of these channels [9] and is therefore a useful tool for investigating the roles of these channels in cognitive processes. These channels are opened by increases in intracellular Ca^2+^. Increases in neural activity principally involve excitatory glutamatergic signaling through AMPA receptors. However, strong glutamatergic stimulation also activates NMDA receptors. Whereas AMPA receptor activation principally increases the movement of Na^+^ ions across the membrane (depolarization), the NMDA receptor activation also allows Ca^2+^ ions to flow into the cell, causing further depolarization. Increased [Ca^2+^]_i_ has many downstream effects, including the activation of transcription factors, that play roles in neuroplasticity. Another effect of NMDA channel activation and Ca^2+^ influx is the opening of sK_ca_ channels, which are hyperpolarizing thus decreasing neuronal activity [9,10].

### 4.2. sK_ca_ Channels in Aging Research

Prior studies have implicated increased sK_ca_ channel activity in cognitive deficits in aging. Aged neurons have less well-regulated and higher cytosolic [Ca^2+^] [3]. High [Ca^2+^]_i_ activates the sK_ca_ channels. Opening the sK_ca_ channels hyperpolarizes the cells, decreasing their activity. The increasing opening of the sK_ca_ channels also increases the duration of the after-hyperpolarization, further decreasing neuronal activity. sK_ca_ channel activation has also been associated with increases in reactive oxygen species (ROS), and overproduction of ROS has been implicated in pathologies of aging, Alzheimer’s Disease, and Parkinson’s Disease [13,14,15,16,17]. Our focus was on the consequences of altered sK_ca_ channel activity on membrane resting potential. Evidence for the involvement of sK_ca_ channels in learning deficits associated with aging comes from an experiment that subjected aged and young rats to fear conditioning involving a sound and then a foot shock. Both young and old rats retained the memory of the association; however, if there was a time delay (1 h) between the sound and the foot shock, only the young rats showed fear responses to the tones when tested one day later. However, if the aged rats were treated with sK_ca_ channel antisense oligonucleotides, they showed the same response to this hippocampal-dependent memory task as the young rats [9]. In other studies, when sK_ca_ channels in hippocampal neurons were inhibited by apamin, excitability of hippocampal neurons increased, LTP improved, and cognitive deficits lessened [18,19]. In Alzheimer’s Disease-affected mice, inhibition of sK_ca_ channels improved cholinergic function and nicotinic excitation [20,21]. Many studies of hippocampal-dependent learning tasks have shown modulating sK_ca_ channel activity is of interest in understanding pathologies of age-related deficits. We add to this important body of knowledge an extension to include a greater variety of cognitive behaviors beyond learning and memory that could be improved by lessening the hyperpolarization of resting membrane potentials.

### 4.3. Different Responses to Apamin Seen in Different Cognitive Dimensions

We showed that apamin effects differ in different cognitive behavioral tests. A common approach in studies of cognition is to focus on tests of learning and memory. However, the formation of long-term memories may not be of value in certain cognitive modes. For example, it would be maladaptive to have to learn to avoid commonly experienced risks. We used exploratory behavior in the open field behavior and in the elevated, open and closed plus maze to assess anxiety and risk avoidance. The only significant result in these experiments was the higher number of “head dips” in baseline. Head dips are seen in the open areas of the plus maze when the animal looks over the edge of the platform. This baseline assessment of risk is followed by avoidance of risk in subsequent trials. Thus, the information obtained by head dips might be considered a form of short-term memory, but it is not influenced by apamin administration.

Homeostatic behaviors such as eating, drinking, and thermoregulation require very little learning in a laboratory ad lib environment. However, these behaviors involve motivation, activity, and motor skills that may show decrements with age and therefore benefit from increased neural activation. We show significant improvements in sucrose preference and in nest-building behavior as outcomes of apamin administration (Figure 4). Poor nest building and decreased attractiveness of the higher concentration sucrose solution have been interpreted as indicators of depression conditions commonly associated with aging. However, the apamin-induced improvement in these measures could be due to increased neural activation, which increases general activity, motivation, and motor skills.

Sociality involves strong learning components—dominance relationships, mating behavior, mate choice—and undergoing aging effects. However, our results show no effects of apamin in the tests of sociability that we conducted (Figure 5). What our data show is a tendency to explore in baseline tests, but then less social behavior following vehicle or apamin administration. An important factor is that these sociality tests were all conducted on aged male/male pairs. We think that following the initial exposure, the major factor in the subsequent tests is a tendency to avoid contacts that could engender aggressive interactions and instead spend more time exploring.

In the results of several of our tests, there were differences (some significant, but most not) between our baseline trials and both the saline and apamin administration trials. We ascribe these differences as being due to the stress of handling for injections.

### 4.4. Positive Apamin Affects Short-Term Memory

Apamin administration had a clear effect on spontaneous alternations in the T-Maze, an indication of short-term memory. We were fortunate to have results from T-Maze testing on these mice when they were young, and therefore we showed a clear decrement associated with aging. That decrement was reversed by the apamin treatment (Figure 3A). Considering the strong effects of apamin on short-term memory, we were surprised by the lack of effect of apamin long-term memory tests. Short-term memory is crucial for even ordinary daily activities. In fact, it can be argued that all the behaviors in this study involved short-term memory, which requires only weak connectivity. NOR and NOL likely depend on stronger connectivity, which is harder to generate in the aged animals. We hypothesize that the apamin injection improved only the encoding of the memory, necessary for all the memory tests we did in this study, but was not enough to improve the consolidation and/or retrieval, necessary for the NOR and NOL.

This work explored the effect of apamin in standard wildtype, non-model, aged mice on general correlates of aging. Our study supports the conclusions of other studies showing that blockers of sK_ca_ channels can alleviate some types of memory deficits and alter some behaviors [19,20,21,22]. What our study adds is evidence that apamin administration can mitigate aging-related impairments of some cognitive functions other than memory.

## 5. Conclusions

Our work demonstrates that apamin improves short-term (working) memory, overall activity, and hedonic behavior in aged mice. Apamin did not affect long-term memory, or risky social behaviors, decision making, or anxiety. The blockage of SK channels should continue to be of interest in the search for novel treatments for aging-related deficits.

## Data Availability

The authors confirm that the data supporting the findings of this study are available within the article.
